# The WWARN Antimalarial Quality Surveyor

**DOI:** 10.1179/204777312X13419245939520

**Published:** 2012-05

**Authors:** P Tabernero, P N Newton

**Affiliations:** 1 Worldwide Antimalarial Resistance Network (WWARN), Centre for Clinical Vaccinology and Tropical Medicine, Churchill Hospital, University of Oxford, United Kingdom; 2 Wellcome Trust-Mahosot Hospital-Oxford University Tropical Medicine Research Collaboration (LOMWRU), Mahosot Hospital, Vientiane, Lao PDR

**Keywords:** Malaria, Medicines, Falsified, Substandard, Mapping

Over the last decade there has been a resurgence of awareness that poor medicine quality is a vitally important, but neglected, public health issue. A growing number of studies have highlighted the importance of drug quality with examples from all over the world. This is an important problem, not only for antimalarials but for all types of medication. The use of poor quality medicines can result in deaths amongst those who would have otherwise survived their infection. They erode public confidence in medicines; have dire economic consequences for patients, their families, health systems and pharmaceutical producers. Of particular relevance are those containing sub-therapeutic amounts of the active pharmaceutical ingredients that can engender drug resistance.

Scientific data on the frequency and consequences of poor quality medicine are relatively sparse. There is no clear picture on the prevalence of poor quality antimalarials, their geography and trading patterns. There are diverse sources of information such as newspaper articles; websites from medicine regulatory authorities (MRAs) and national malaria control programmes (NMCPs), and academic publications, but there is no single accurate global databank, with a mapping system of publically-accessible detailed information on poor quality antimalarials.

In order to try to fill this gap, the WorldWide Antimalarial Resistance Network (WWARN; www.wwarn.org) has built a new Antimalarial Quality Scientific Group that aims to collate information and stimulate discussion on the epidemiology of poor quality antimalarials. The work conducted by the Scientific Group includes a database and an interactive web application displaying the spread of poor quality antimalarials through space and time, the AQ Surveyor (see Fig. 1 and http://www.wwarn.org/aqsurveyor/). We hope that this will help enhance information availability, increase understanding, monitor geographical and temporal trends, and be especially useful for medicine regulatory authorities and malaria control programmes. This work also aims to facilitate improvements both in sampling and analytical methodology, and advocate for more investment in this, but neglected, aspect of public health.

**Figure 1:**
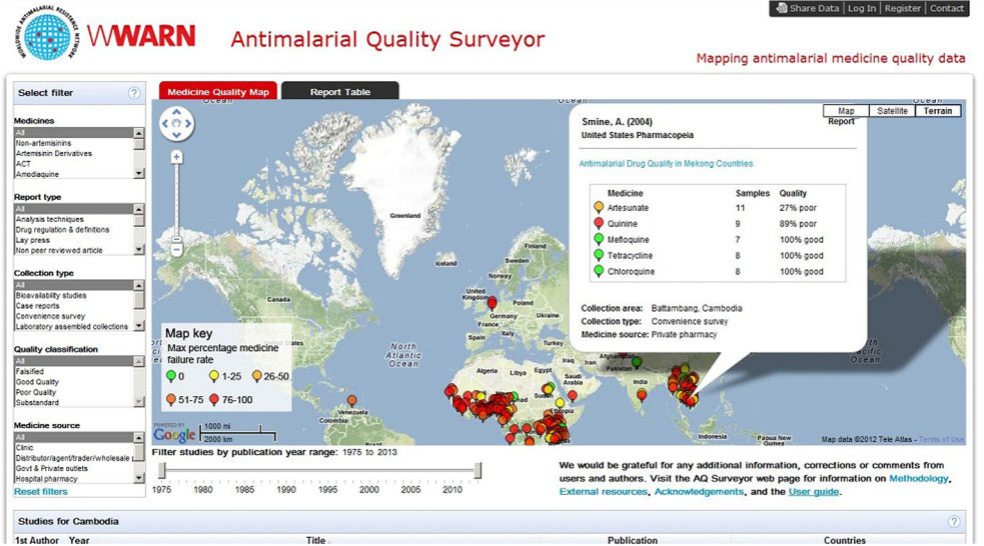
Web screenshot of the Antimalarial Quality Surveyor.

## Antimalarial Medicine Quality Surveyor

A systematic review of scientific and lay reports was conducted, including articles from news agencies on antimalarial medicine quality. Abstracts and full text of more than 200 studies were reviewed, and information about each publication, the data within and their interpretation were entered into a comprehensive database.

The Antimalarial Medicine Quality Surveyor (Fig. 1) was built using Google’s Web toolkit and Google Maps. The information from each publication can be viewed after selecting from a set of filters:

- **Medicine**: allows the user to select the medicines, based on *Internationa Nonproprietaryl* *Names* (INN) or categories of medicines.
- **Report type**: Lists the different types of publications found describing antimalarial drug quality.
- **Collection type**: Describes how the collection of antimalarial medicines was conducted in each report.
- **Quality classification**: defines the different types of poor quality medicines reported, such as falsified and substandard
- **Outlet type/Medicine Source**: describes the type of outlet where samples were collected
- **Date**: which can be used to restrict the year of publication of the report

The AQ Surveyor displays one map plus a tabular display of details of the underlying publications. Each map contains pushpin markers of different colours identifying places from where antimalarial quality data was collected and the pin colour represents the medicine failure rate reported in the publication.

Further ‘drill down’ information can be obtained by clicking on the pins. This information includes the details of the publication, a hyperlink to an abstract or full paper, and details of the methods and findings. A tabular view is provided, at the right, listing all the source reports plus other publications, such as reviews and descriptions of assay techniques, without primary data amenable to mapping, but relevant to medicine quality.

Reliable data are required to inform responses and prioritize interventions. These results illustrate the alarming scale of the problem in many developing countries, but also highlight major geographical gaps in the assessment of this public health emergency and the poor quality of many of the data. We hope that this will provide evidence to help improve medicine quality, inform improvements in medicine regulation and to contain and impede antimalarial resistance.

